# Systematic Mapping of Research on Vaccine-Preventable Diseases in Children in Sub-Saharan Africa: A Decennial Scientometric Analysis

**DOI:** 10.3390/vaccines11091507

**Published:** 2023-09-21

**Authors:** Chidozie Declan Iwu, Chinwe Iwu-Jaja, Anelisa Jaca, Charles Shey Wiysonge

**Affiliations:** 1 School of Health Systems and Public Health, Faculty of Health Sciences, University of Pretoria, Pretoria 0001, South Africa; 2 Cochrane South Africa, South African Medical Research Council, Tygerberg, P.O. Box 19070, Cape Town 7505, South Africa; chinwelolo@gmail.com (C.I.-J.);; 3 World Health Organization Regional Office for Africa, Cité du Djoué, Brazzaville P.O. Box 06, Congo

**Keywords:** vaccine preventable diseases, VPDs, bibliometric analysis, scientometric analysis, Africa

## Abstract

Vaccine-preventable diseases (VPDs) remain a significant public health challenge, particularly in sub-Saharan Africa. The high burden of VPDs in this region necessitates the need for continued investigation and intervention. This paper presents a bibliometric analysis of research on VPDs in children in sub-Saharan Africa in the last 10 years to capture the current state of research in the field. This study used a systematic search for articles published between 2013 and 2022 in the Web of Science Core Collection database and, subsequently, scientometric techniques for data analyses and interpretation. Annual scientific production of publications on the research of VPDs in children in sub-Saharan Africa increased from 2013 to 2019 and then gradually declined. South Africa had the most VPD studies (*n* = 148; 16.2%), followed by Nigeria, Ghana, Kenya, The Gambia, Malawi, Ethiopia, and the Republic of Congo. The *Vaccine* journal published the most. The *Pan African Medical Journal* was the most frequent destination journal based in Africa. The commonly studied pathogens were *Streptococcus pneumoniae* and *Haemophilus influenzae*. Research productivity increased exponentially in the pre-COVID era and declined in the past two years, so more VPD research in this region is needed.

## 1. Introduction

Vaccine-preventable diseases (VPDs) continue to pose a significant burden on children in sub-Saharan Africa, with over thirty million children under five affected in Africa [1,2,3]. Based on a 2018 publication, more than half a million children die from VPDs yearly in Africa, with this figure representing 58% of all global deaths [2].

Despite significant progress in the introduction of vaccines into routine immunization programmes and improved vaccination coverage in recent years [3], VPDs among children still remain a threat. These diseases, including infectious diseases such as measles, polio, and rotavirus, can have serious health consequences, including death. Furthermore, routine immunization services faced disruptions due to the COVID-19 pandemic, which may have resulted in a further decline of vaccination coverage [4].

Several factors contribute to the high burden of VPDs in sub-Saharan Africa, including inadequate vaccination coverage, poor access to health care, and underlying socioeconomic and environmental conditions [2,5]. To address this burden and improve the health of children in the region, it is important to understand the current state of knowledge about VPDs in sub-Saharan Africa and identify the research gaps. 

To the best of our knowledge, a comprehensive systematic analysis of VPD research in sub-Saharan Africa is yet to be conducted [6]. Such an analysis would provide a valuable resource for researchers and policymakers working to reduce the burden of VPDs in the region.

Therefore, we conducted a systematic research mapping of common VPDs in children in sub-Saharan Africa, with the aim of identifying the current state of knowledge and identifying gaps in research. By understanding the existing research and identifying areas where further investigation is needed, we hope to contribute to efforts to reduce the burden of VPDs on children in sub-Saharan Africa.

## 2. Materials and Methods

### 2.1. Study Design

We used the systematic scientometric technique to appraise the research outputs of VPDs, quantitatively and qualitatively, in children in sub-Saharan Africa in the last 10 years using the method described by Aria et al. [7]. This period is likely to reflect the most recent advancements in vaccination strategies, changes in disease burden, and evolving research priorities, which can have direct implications for public health interventions.

This study design, consisting of a bibliometric index analysis (BIA) and a scientific network map analysis (SNMA), is invaluable for comprehensively exploring the existing knowledge base, intellectual structure, and emerging trends of any research niche. It is also useful for assessing the conceptual structure of a given research area and produces social networks of a scientific community [7].

### 2.2. Data Source

Relevant research publications on VPDs in children in sub-Saharan Africa were retrieved from the Web of Science (WoS) core collection database and its indices, including the following: Science Citation Index Expanded (SCI-EXPANDED); Social Sciences Citation Index (SSCI); Arts and Humanities Citation Index (A&HCI); Conference Proceedings Citation Index—Science (CPCI-S); Conference Proceedings Citation Index—Social Science and Humanities (CPCI-SSH); Book Citation Index—Science (BKCI-S); Book Citation Index—Social Sciences and Humanities (BKCI-SSH); and Emerging Sources Citation Index (ESCI). This database was used because it is a repository of comprehensive and multidisciplinary citation data and has emerged as a conventional data source for bibliometric analysis [8]. Additionally, WoS provides the substantial bibliometric indicators necessary for scientometric analysis [8]. 

### 2.3. Data Search and Retrieval

The WoS database was searched for relevant publications on 10 January 2023, 15:00 Greenwich Meridian (GMT), using the “title search” strategy. This strategy was used because of its ability to pull substantial relevant publications with increased sensitivity and specificity [9]. The search and retrieval were conducted following the preferred reporting items for systematic reviews and meta-analyses (PRISMA) approach [10]. A combination of comprehensive search terms made up of VPDs terms (“vaccine preventable disease*” OR “vaccine-preventable disease*” OR diphtheria OR “Corynebacterium diphtheriae” OR tetanus OR “Condyloma accuminata” OR polio OR poliovirus OR poliomyelitis OR pertussis OR “whooping cough” OR “Bordetella pertussis” OR “Haemophilus influenzae type b” OR “hepatitis B” OR pneumococcal OR “Streptococcus pneumoniae” OR meningococcal OR meningococcus OR “Neisseria meningitidis” OR mumps OR measles OR rubella OR “Human papillomavirus” OR HPV OR influenza OR flu OR “hepatitis A” OR shingles OR Varicella OR chickenpox OR typhoid OR Salmonella OR Rotavirus OR cholera OR “Vibrio cholerae” OR “yellow fever” OR rabies OR lyssaviruses OR “Japanese encephalitis” OR leptospirosis OR Leptospira) AND children terms (child* OR children OR infant* OR infancy OR pediatric* OR juvenile*) were used. The VPDs terms were adapted from the literature [11]. The search was refined by publication years (2013 to 2022), countries/regions (individual sub-Saharan African countries), document types (research article), and language (English). The data from WoS were retrieved in a Bibtex format and imported into the Mendeley referencing software, v1.19.8, where they were validated for relevance by another author (C.I.-J.). We excluded tuberculosis because a bibliometric analysis on TB in sub-Saharan Africa has just been recently published [12]. 

### 2.4. Data Analysis

Descriptive data analysis including visualization and contextual scientific mapping of the retrieved data was conducted using the biblioshiny application of the bibliometrix package in RStudio (version 4.2.2). The bibliometric parameters, including sources, authors, documents, keywords, conceptual structure, intellectual structure, and social structure, were analyzed. To produce informative yet visible scientific network maps, we stuck to the following method parameters: automatic network layout, Walktrap clustering algorithm, 30–50 nodes, 0.1 repulsion force, 4 minimum number of edges, and removed isolated nodes. We only used research articles for our analysis. We also assessed the following indicators: “major sources of research publications” on VPDs in children in sub-Saharan Africa, which refer to the databases or sources of academic literature that are considered pertinent to the research question or topic being studied. These sources typically include academic journals, conference proceedings, books, and other scholarly publications. In this study, we considered only academic journals. We assessed “the most cited documents” by analyzing the most cited publications both globally and locally. Globally, the most cited publications are those that have received the most citations globally, whereas locally, the most cited publications are those that have received the most citations within Africa. The “conceptual, intellectual, and social structures” of VPD research in children in Sub-Saharan Africa from 2013 to 2022 were also evaluated. The theoretical frameworks and concepts that researchers use to guide their work are referred to as conceptual structures. For example, researchers may employ concepts such as herd immunity, vaccine efficacy, or vaccine hesitancy. The patterns of thought and reasoning that underpin field research are referred to as intellectual structures. This could include specific methodological approaches, modes of analysis, or data interpretation methods. The relationships and interactions among researchers and research groups in the field are referred to as social structures. Collaborations between researchers, patterns of authorship and citation, or the formation of research communities around specific topics or approaches are examples of this. Lastly, we assessed keywords and trending topics on VPDs in children in sub-Saharan Africa. 

### 2.5. Ethical Consideration

This study involved a review of publicly available literature. Ethical approval is not required as neither people nor animals were directly involved in the study design, conduct, or reporting.

## 3. Results

### 3.1. Search Results 

**Figure 1:**
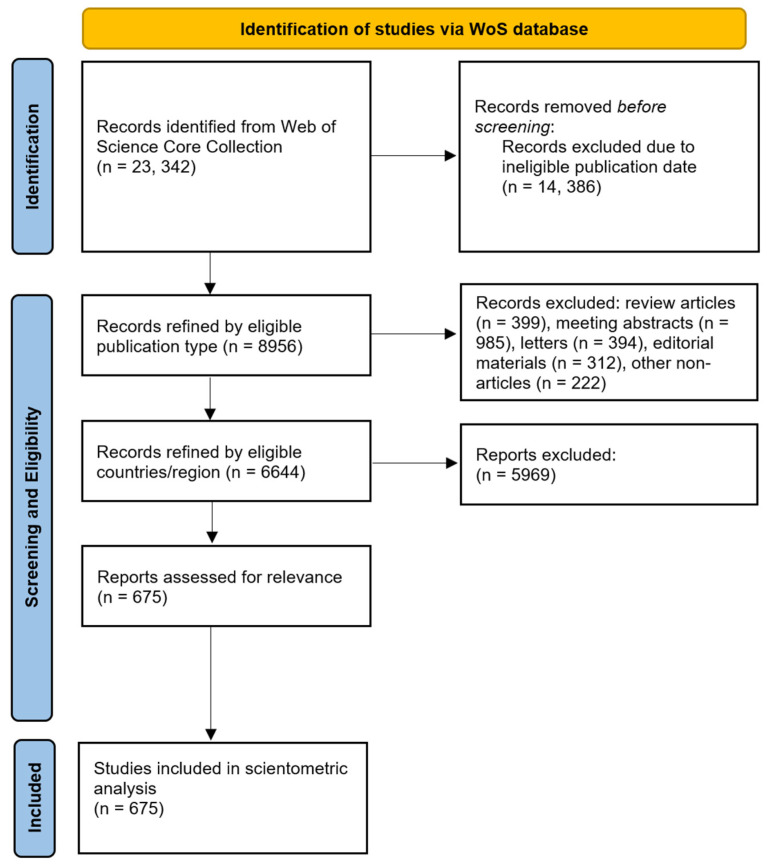
The PRISMA flow diagram of the study selection process.

A total of 23,342 publications were identified from the literature search, from which 14,386 were excluded as they were not published between 2013 and 2022. The remaining 8956 publications were refined by article type and their status as original articles, and 6444 publications were retained. These publications were further refined by the country/region the study was conducted, and only studies conducted in sub-Saharan Africa (*n* = 675) were retained. These eligible publications were further validated and included in the final analysis, as shown in the PRISMA flow diagram (Figure 1).

### 3.2. The Characteristics of the Included Studies

The characteristics of the publications on VPDs in children in sub-Saharan Africa (2013–2022) are shown in Table 1. Of the 675 articles retrieved from 170 sources, 672 were research articles, 1 was a data paper, and 2 were early access. Between 2013 and 2022, the average growth rate of research publications was 4.71%, the average citations per document was 12.69; the total references were 15,250, the total keywords were 1270, and the author’s keywords observed were 1213. These publications were written by 4658 authors during this period, of which only three single-authored documents were observed. About 10 co-authors in each co-authored document were observed. 

**Figure 2:**
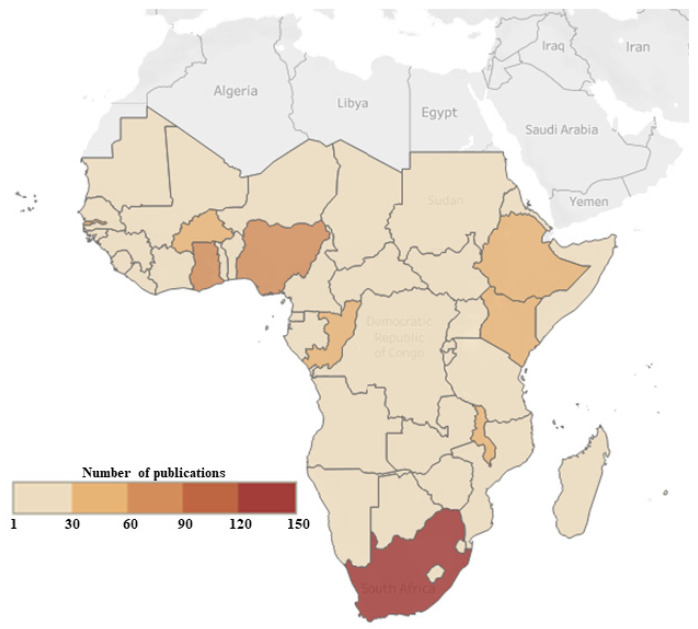
The geographical distribution of the research on VPDs in children in sub-Saharan Africa (2013–2022).

The geographical distribution of the research on VPDs in children in sub-Saharan Africa is shown in Figure 2. Most of the studies were conducted in South Africa (*n* = 148), followed by Nigeria (*n* = 75) and Ghana (*n* = 64) and then Kenya (*n* = 59), Gambia (*n* = 55), Malawi (*n* = 52), Ethiopia (*n* = 51), and the Congo (*n* = 50).

**Figure 3:**
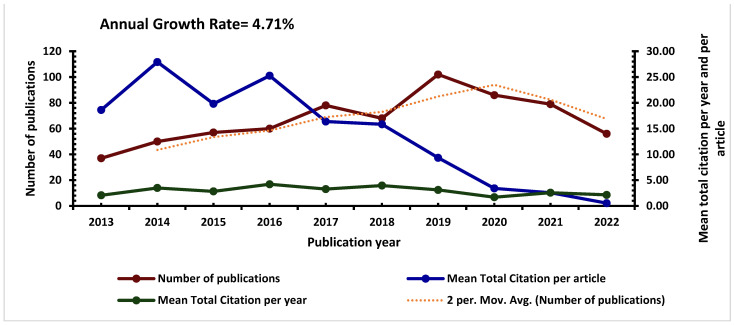
The annual distribution, mean total citation per year, mean total citation per article, and moving average (Mov. Avg) of the annual publications on vaccine-preventable diseases in children in sub-Saharan Africa (2013–2022).

As shown in Figure 3, there was a steady increase in the overall annual scientific production (number of publications, mean total citation per article, and mean total citation per year) of research publications on VPDs in children in sub-Saharan Africa from 2013, but this dropped from 2020 to 2022. However, the mean total citations per year were relatively unchanged between 2013 and 2022. 

### 3.3. Major Sources of Research Publications on VPDs in Children in Sub-Saharan Africa

The top 10 major sources of publications on VPDs in children in sub-Saharan Africa between 2013 and 2022 are shown in Table 2. *Vaccine* was found to be the major source on this subject, with about 67 publications and 1443 local citations during the study period. Based on the number of publications, other sources, such as *Clinical Infectious Diseases*, *PLOS One*, the *Pediatric Infectious Disease Journal*, the *Journal of Infectious Diseases*, *BMC Infectious Diseases*, *BMC Public Health*, the *American Journal of Tropical Medicine and Hygiene*, the *Pan African Medical Journal*, and *Human Vaccines and Immunotherapeutics*, were found to be prominent. In addition to these sources, and based on the number of local citations, *The Lancet*, the *Journal of Clinical Microbiology*, *Lancet Infectious Diseases*, and *The New England Journal of Medicine* were also found to be prominent. These sources were found to have a high source impact. For instance, *Clinical Infectious Diseases* had a 22 H-Index and a 34 G-Index, while *PLOS One* had a 15 H-Index, a 22 G-Index, and a 1.5 M-Index. 

### 3.4. The Most Cited Documents 

**Figure 4:**
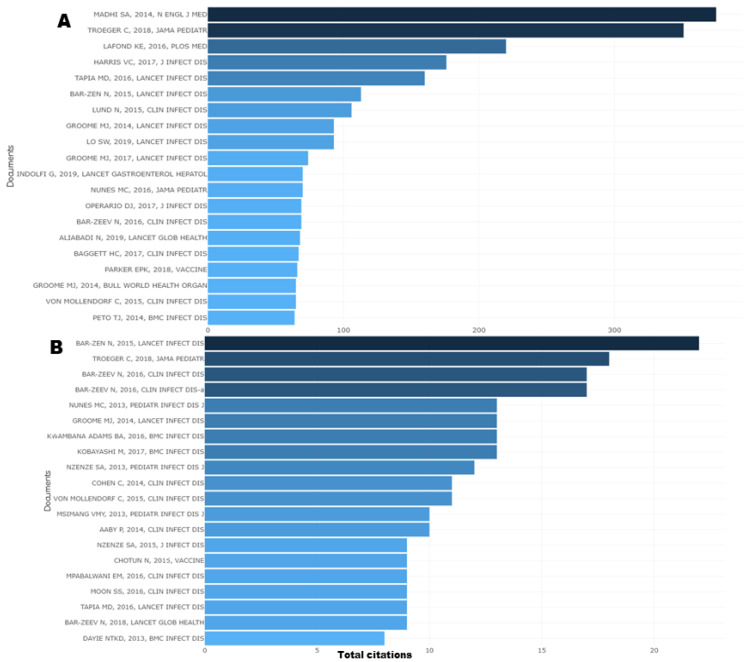
Most-cited publications on VPDs in children in sub-Saharan Africa, globally (**A**) and locally (**B**), from 2013 to 2022.

Figure 4 shows the most-cited publications on VPDs in children in sub-Saharan Africa, globally and locally, from 2013 to 2022. Global citation counts all the citations a given article received all over the world, while local citation counts the citations received by a reference article “internally to your collection” [7]. Among the globally most cited publications, Madhi et al. (2014) [13] was the most cited during the study period, with about 375 total citations. This study was conducted in South Africa to evaluate the efficacy of the trivalent inactivated influenza vaccine in HIV-infected and HIV-uninfected pregnant women and their infants. It was found that the vaccine was immunogenic in HIV-uninfected and HIV-infected pregnant women and provided partial protection against confirmed influenza in both groups of women and in infants who were not exposed to HIV. The most locally cited document was Bar-Zen (2015) [14], with 22 local citations and 113 global citations. This study was conducted in Malawi to assess the impact and effectiveness of the monovalent human rotavirus vaccine on rotavirus gastroenteritis in infants. It was found that the routine use of rotavirus reduced hospital admissions for several genotypes of rotavirus in children younger than 5 years. 

### 3.5. Corresponding Author’s Affiliations and Overall Country Scientific Production

**Figure 5:**
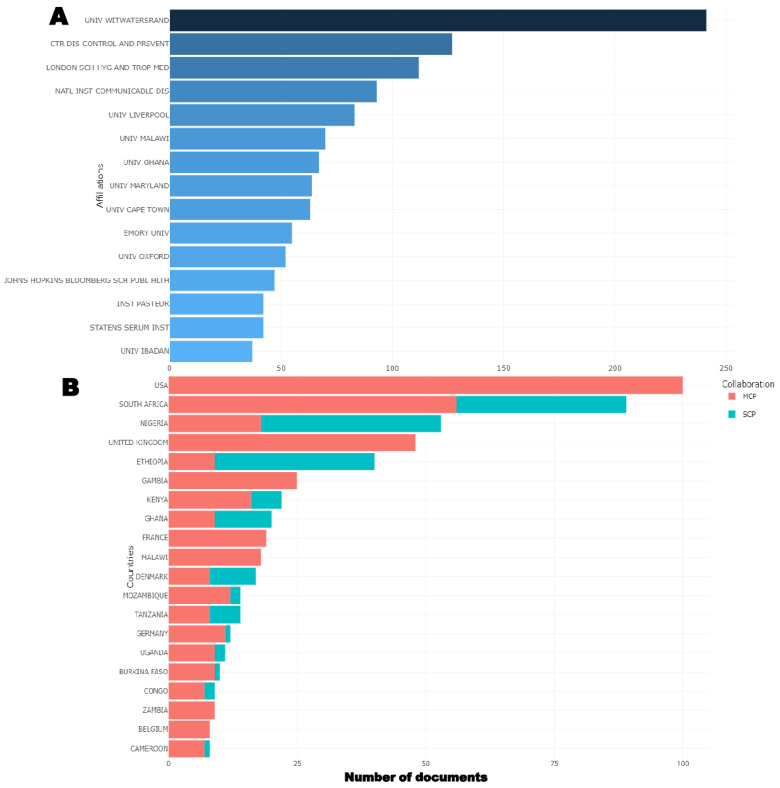
Prominent author’s affiliations (**A**) and corresponding author’s country (**B**). SCP: single-country publications; MCP: multiple-country publications.

The top 15 affiliations and countries of the corresponding authors involved in VPDs research in children in sub-Saharan Africa are shown in Figure 5. University of the Witwatersrand was the most prominent affiliation, with 241 publications during the study period. This was followed by the Centre of Disease Control and Prevention, the London School of Hygiene and Tropical Medicine, and the National Institute for Communicable Diseases, with 126, 112, and 93 publications, respectively, as shown in Figure 5A.

Based on the corresponding author’s country, the USA had the highest number of publications (*n* = 100), all of which were multiple-country publications (MCP), as shown in Figure 5B. This was followed by South Africa, with 89 publications, broken down into 56 MCP and 33 single-country publications (SCP); Nigeria, with 53 publications, 18 MCP and 35 SCP; and the United Kingdom, with 48 publications, 48 MCP and 0 SCP.

**Figure 6:**
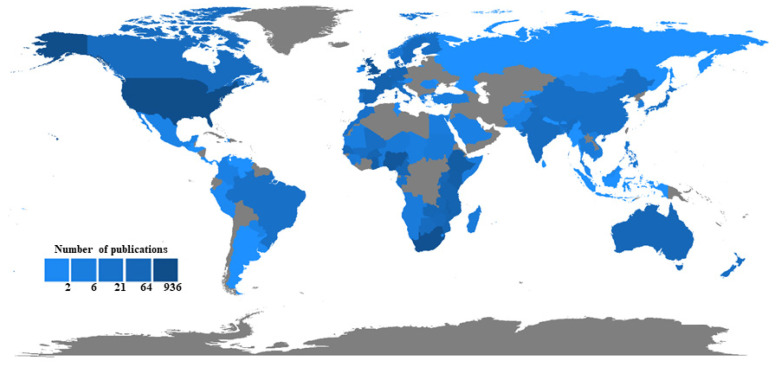
Global appearances of authors on VPDs in children in Sub-Saharan Africa by country of affiliation 2013–2022.

Based on the overall country scientific production, the number of “authors’ appearances by country of affiliations” were analyzed and shown in Figure 6. This analysis takes into account that each article may have multiple authors from different countries, and thus contributes to the scientific output of each of those countries. The number of appearances of each author from a given country is counted separately, so that the total number of appearances for that country is the sum of all authors’ appearances on all articles. Hence, the number of publications in the overall country scientific research production exceeded the number of articles (*n* = 675) initially included in this study. Based on this analysis, the USA ranked first, with about 935 publications. Other highly productive countries were South Africa (*n* = 641), the United Kingdom (*n* = 479), Nigeria (*n* = 349), Ethiopia (*n* = 183), Kenya (*n* = 164), Ghana (*n* = 161), Malawi (*n* = 157), and France (*n* = 109). 

### 3.6. Keywords and Trending Topics on VPDs in Children in Sub-Saharan Africa, 2013–2022

**Figure 7:**
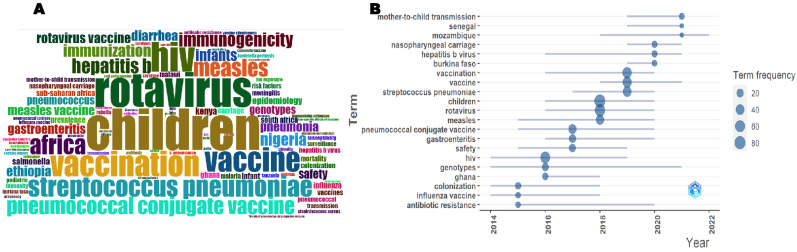
A word cloud (**A**) and trend topics (**B**) on VPDs in children in sub-Saharan Africa, 2013–2022.

A word cloud showing the most prominent used words (top 100) based on their frequency of occurrence and the trend topics on VPDs in children in sub-Saharan Africa, 2013–2022, is shown in Figure 7. The word cloud displays, at a glance, the most used words from small to large according to how often they appeared in the publications, as shown in Figure 7A. In this study, the most used words were “rotavirus”, “HIV”, “vaccination”, “*Streptococcus pneumoniae”*, “Pneumococcal conjugate vaccine”, “Africa”, “measles”, “hepatitis B”, and “vaccine”. Putting these words together implies that most research on VPDs in children in sub-Saharan Africa during the study period is focused on rotavirus, measles, hepatitis B, HIV, vaccines, and vaccination. These terms were shown to be heavily used in 2018 and 2019, as shown in Figure 7B. Prior to this period, words such as “gastroenteritis” and “safety” also appeared frequently in the publications. Between 2020 and 2022, words/phrases such as “mother-to-child transmission”, “Senegal”, “Mozambique”, and “nasopharyngeal carriage” also appeared frequently. 

### 3.7. Conceptual, Intellectual, and Social Structures of Research on VPDs in Children in Sub-Saharan Africa, 2013–2022

**Figure 8:**
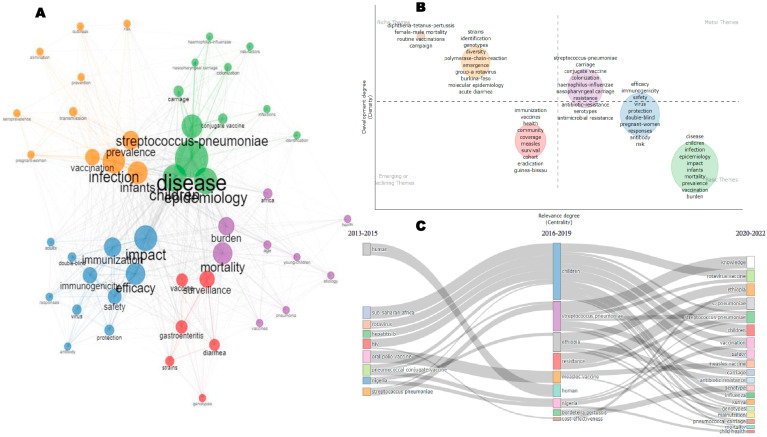
The conceptual structure of VPDs in children in sub-Saharan Africa (2013–2022) showing the co-occurrence network (**A**), thematic map (**B**), and thematic evolution (**C**).

The conceptual structure of research on VPDs in children in sub-Saharan Africa, 2013–2022, presented by a co-occurrence network, a thematic map, and three-staged thematic evolution, is shown in Figure 8. In the keyword co-occurrence network (Figure 8A), rotavirus significantly co-occurred with diarrhea, gastroenteritis, genotypes, vaccines, prevalence, and Burkina Faso. Vaccine and Africa significantly co-occurred with immunogenicity, safety, infants, rotavirus vaccine, and hepatitis virus. “Children” significantly co-occurred with “malaria”, “measles” “vaccine”, “Salmonella”, “immunity”, “influenza”, “Ethiopia”, “South Africa”, and “mortality”. “Vaccination” significantly co-occurred with “hepatitis B”, “measles”, “immunization”, “surveillance”, “transmission”, “mother-to-child-transmission”, “epidemiology”, and “Nigeria and Kenya”. “HIV” significantly co-occurred with “conjugate vaccine”, “pneumonia”, “*Streptococcus pneumoniae*”, and “risk factors”. From the thematic map (Figure 8B), four themes were identified. The “niche theme” was made up of words that basically involved identification techniques such as polymerase chain reaction and molecular epidemiology. The “motor themes” was made up of microbial inclined words such as “*Streptococcus pneumoniae*”, “carriage”, “conjugate” “vaccine”, “colonization”, “*Haemophilus influenzae”*, “nasopharyngeal carriage”, “efficacy”, “immunogenicity”, and “safety”. The “basic theme” contained words including “burden”, “epidemiology”, “prevalence”, “vaccination”, and “antimicrobial resistance”, amongst others. “Emerging or declining themes” consisted of words such as “immunization”, “vaccines”, “eradication”, “survival”, “cohort”, “eradication” and “Guinea-Bissau”. Other words, such as “efficacy”, “immunogenicity”, “safety”, etc. intersected between “motor” and “basic themes”. From the thematic evolution map (Figure 8C), *Streptococcus pneumonia* evolved in the three time periods (2013–2015, 2016–2019, and 2020–2022). 

**Figure 9:**
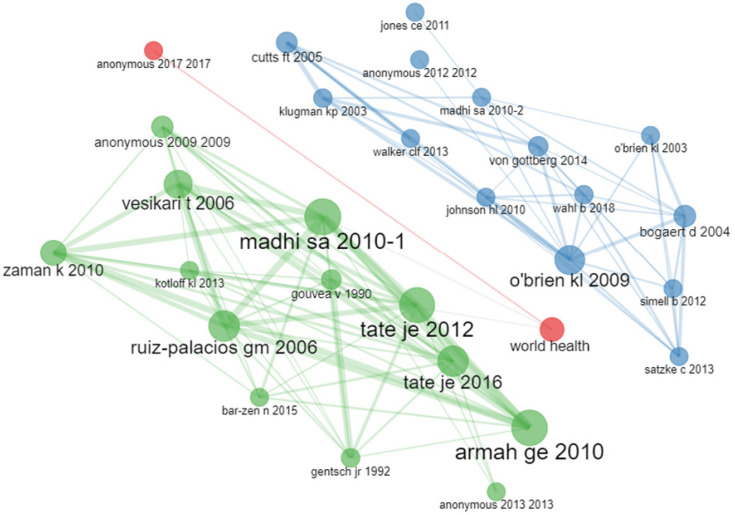
Intellectual structure of VPDs in children in sub-Saharan Africa (2013–2022) based on the co-citation network using a clustering algorithm.

The intellectual structure of research on VPDs in children in sub-Saharan Africa, 2013–2022, presented as a co-citation network using a clustering algorithm, is shown in Figure 9. This analysis was carried out to show the relationship between articles that are cited together. Based on the size of the nodes and the strength of the network, there was a strong co-citation network between Madhi SA (2010-1) and Tete JE (2012) and Tete JJE (2016) and Armah GE (2010) on the one hand; and between O’brien KI (2009), Johnson HI (2010), Bogaert D (2004), amongst others, on the other hand.

**Figure 10:**
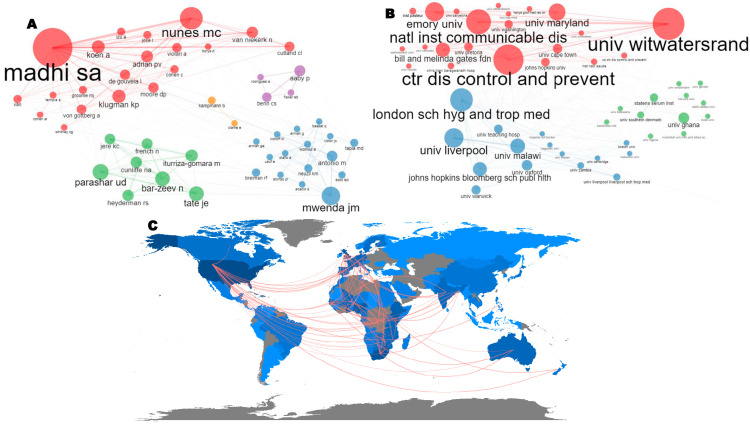
Social structure of VPDs in children in sub-Saharan Africa (2013–2022) showing the author collaboration network (different colours represents different clusters) (**A**), institution collaboration network (different colours represents different clusters) (**B**), and country collaboration network (red lines) (**C**).

A multi-level social structure of research on VPDs in children in sub-Saharan Africa, 2013–2022, presented via an author collaboration network, institution collaboration network, and country collaboration map ,is shown in Figure 10. At the level of author collaboration (Figure 10A), five major collaboration networks were observed in descending order. In one network, Madhi SA significantly collaborated with Nunes MC and other authors such as Klugman KP, Joen A, and Adrian PV. In the second network, a major collaboration was between Mwenda JM and other authors. In the third network, a significant collaboration was observed between Parashar UD and other authors. Aaby P collaborated with Benn CS, Fisker AB, and Rodrigues A in the fourth network, and Kapmann B collaborated with only Clarke E in the fifth network. At the level of institutional collaboration (Figure 10B), three major collaboration networks were observed. In one network, the major collaboration was between the University of the Witwatersrand, the Centre for Disease Control and Prevention, the National Institute for Communicable Diseases, Emory University, and the University of Maryland. A significant collaboration was observed between the London School of Hygiene and Tropical Medicine, the University of Liverpool, the University of Malawi, and John Hopkins Bloomberg School of Public Health in the second network. In the third network, the University of Ghana significantly collaborated with the University of Southern Denmark and the Statens Serum Institute. Concerning country-level collaboration (Figure 10C), the USA significantly collaborated with African countries such as Kenya, Malawi, and Mozambique, as well as other high-income countries such as Australia and Switzerland. Additionally, there was a significant collaboration between African countries such as South Africa, Ghana, and Tanzania and other high-income countries such as Denmark and China. The United Kingdom also collaborated with Gambia, Nigeria, Uganda, Belgium, Germany, and France, amongst others. 

## 4. Discussion

Vaccine-preventable diseases (VPDs) continue to plague sub-Saharan Africa’s children. This paper presents a systematic bibliometric analysis of research on VPDs in children in sub-Saharan Africa from 2013 to 2022.

First, there were a significant number of research articles published on this topic, with 672 out of the 675 articles retrieved being research articles. This highlights the importance of studying VPDs in children in sub-Saharan Africa, which is likely due to the high burden of VPDs in our region [15]. Second, the average growth rate of research publications was 4.71%, indicating a steady increase in research output on this topic over the years. This is a positive sign that research on VPDs in children in sub-Saharan Africa is continuing to grow, potentially leading to improved prevention and treatment strategies in the future.

Third, the average number of citations per document was 12.69, indicating that these publications have been cited frequently in other research. This suggests that the research on VPDs in children in sub-Saharan Africa has had an impact on the wider scientific community and has been recognized as important and valuable. Fourth, the total number of references was 15,250, which highlights the extensive amount of research that has been conducted on this topic. This suggests that there is a large body of knowledge available on VPDs in children in sub-Saharan Africa which can be used to inform future research and interventions. Fifth, the observation that only three single-authored documents were observed suggests that research on VPDs in children in sub-Saharan Africa is often conducted collaboratively, with many co-authors involved in each document. This is likely due to the multidisciplinary nature of the topic, which requires expertise from multiple fields. Finally, the large number of author’s keywords observed (1213) and total keywords (1270) suggests that there are many different aspects and perspectives that are being explored in research on VPDs in children in sub-Saharan Africa. This highlights the complexity of the topic and the need for a multidisciplinary approach in order to address it effectively. Based on the geographical distribution of research on VPDs in children in sub-Saharan Africa, it is clear that South Africa, Nigeria, Ghana, Kenya, Gambia, Malawi, Ethiopia, and the Congo are the countries where most of the research has been conducted. This may be due to several factors, including the availability of research funding, existing research infrastructure, and the burden of VPDs in these countries.

The decrease in the number of publications in 2020–2022 may be attributed to the COVID-19 pandemic, which may have affected research output globally. During this period, researchers and scientific resources were redirected toward addressing the urgent and pressing challenges posed by the pandemic. Governments, funding agencies, and institutions worldwide shifted their priorities towards COVID-19 research, potentially leading to a decrease in research efforts on other topics, including VPDs. Additionally, the pandemic-induced travel restrictions, lockdowns, and disruptions to daily life might have hindered researchers’ ability to conduct fieldwork, gather data, or collaborate effectively. This could have affected the production of new research publications, particularly in regions such as sub-Saharan Africa, where access to resources and research infrastructure might already be limited. Moreover, the drop in research output could be influenced by delays in the publication process. Researchers might have faced challenges in completing their studies, submitting manuscripts, and going through the peer-review process due to disruptions caused by the pandemic. Despite the decrease in the number of publications, the mean total citations per year has remained relatively unchanged over the years. This could be due to the continued relevance and impact of existing research on VPDs in children in sub-Saharan Africa. To address the downward trend in research output and mean total citations per article, there is a need for increased research funding, improved research infrastructure, and enhanced collaboration between researchers and policy-makers. Additionally, efforts should be made to improve the accessibility and dissemination of research findings to ensure that they reach the relevant stakeholders and inform policy decisions.

The results of the analysis of the top 10 major sources of publications on VPDs in children in sub-Saharan Africa between 2013 and 2022 are informative. The findings indicate that the *Vaccine* journal was the major source in this subject, with about 67 publications and 1443 local citations during the study period. This suggests that *Vaccine* is a highly influential source in the field of VPDs in children in sub-Saharan Africa.

The prominence of other sources such as *Clinical Infectious Diseases*, *PLOS One*, the *Pediatric Infectious Disease Journal*, the *Journal of Infectious Diseases*, *BMC Infectious Diseases*, *BMC Public Health*, the *American Journal of Tropical Medicine and Hygiene*, the *Pan African Medical Journal,* and *Human Vaccines and Immunotherapeutics*, based on the number of publications, is noteworthy. This suggests that these sources have been instrumental in disseminating research findings on VPDs in children in sub-Saharan Africa. In addition to these sources, *The Lancet*, the *Journal of Clinical Microbiology*, *Lancet Infectious Diseases*, and *The New England Journal of Medicine* were also found to be prominent based on the number of local citations. The high source impact of *Clinical Infectious Diseases* and *PLOS One*, as indicated by their H-Index, G-Index, and M-Index, reflects the productivity and impact of these journals in a particular field. The high H-Index and G-Index of *Clinical Infectious Diseases* and *PLOS One* suggest that they are highly influential sources in the field of VPDs in children in sub-Saharan Africa. Overall, the findings of this analysis suggest that there are several influential sources of publications on VPDs in children in sub-Saharan Africa, and that these sources have been instrumental in disseminating research findings on this important subject. Researchers and policy-makers can use this information to identify the most major and influential sources of information on VPDs in children in sub-Saharan Africa.

The high number of publications from the CDC and the London School of Hygiene and Tropical Medicine reflects the important role that international organizations play in conducting research on VPDs in Africa. This is particularly important given the global nature of vaccine-preventable diseases and the need for coordinated efforts to combat them. 

The fact that the USA had the highest number of publications, all of which were multi-country publications, is not surprising given the country’s significant investment in global health research and development. Furthermore, there could be several reasons why researchers from the USA are more involved in research on VPDs in Africa, of which one possible reason is funding. The USA is a major contributor to global health funding, and some of this funding may be directed towards research on VPDs in Africa. In addition, some US-based research institutions and universities may have established collaborations or partnerships with African institutions, which may lead to increased research activity in the region. However, it is important to note that there may be other factors at play as well, and more research would be needed in order to fully understand the reasons behind the observed patterns.

The co-occurrence network, thematic map, and thematic evolution presented in Figure 8 provide an insightful view of the conceptual structure of research on VPDs in children in sub-Saharan Africa from 2013 to 2022. The keyword co-occurrence network in Figure 8A highlights the most frequently co-occurring words in the publications during the study period. The network reveals that “rotavirus” is a significant research topic, and it is associated with “diarrhea”, “gastroenteritis”, and “vaccines”. “Vaccination” is frequently associated with “hepatitis B”, “measles”, and “mother-to-child transmission”, highlighting that the studies were focused on vaccination.

The thematic evolution map in Figure 8C shows how research themes have evolved. The map indicates that “*Streptococcus pneumoniae*” is a persistent research topic across all three time periods, suggesting its continued importance in the region. The emergence of words such as “immunization” and “vaccines” as dominant themes in the later periods is consistent with the increased focus on vaccination in the region. Possible explanations for the findings could include a growing interest in vaccination as a means of preventing VPDs, particularly given the burden of disease in the region. *Streptococcus pneumoniae* is a significant research topic due to its high prevalence and association with severe disease in children. The persistence of research on *Streptococcus pneumoniae* in the region may be related to the continued burden of disease and the need for effective prevention and treatment strategies.

## 5. Study Limitations and Strengths

First, the scientometric analysis was conducted using the WoS-indexed publications only. This introduces the possibility of our missing relevant publications that are not in the WoS core collections. However, the WoS database has a reputation for providing huge amounts of bibliometric indicators required for scientometric analysis [16]. 

Second, refining the search to English language only and articles only may have slightly reduced the generalizability of the findings. However, this limitation may be negligible as 100% of the papers finally retrieved were written in English Language. Additionally, 95% of publications in Google Scholar are written in English language [17]. 

## 6. Conclusions

The findings of this study suggest that research on VPDs in children in sub-Saharan Africa is diverse and complex, with several key themes emerging, including vaccine efficacy and safety, disease burden and epidemiology, and antimicrobial resistance. There has been a steady increase in the overall annual scientific production of research in the last decade, with most studies conducted in South Africa and the majority being published in the *Vaccine* journal. *Streptococcus pneumoniae* and *Haemophilus influenza* were the most studied pathogens, and collaboration among authors, institutions, and countries were found to be critical to advancing knowledge on this topic. Our analysis highlights the need for continued research on VPDs in this region, particularly in areas such as immunization and vaccines. Considering the variety of VPDs, we recommend more primary studies to focus on a broader range of pathogens. Further exploration of country-specific data could be beneficial to contextualize the results and aid in formulating targeted intervention strategies. By addressing these gaps, we can work towards reducing the burden of VPDs on children in sub-Saharan Africa and improving global health outcomes.

## Figures and Tables

**Table 1:** Summary of the data on vaccine-preventable diseases in children in sub-Saharan Africa (2013–2022).

Description	Results
Main information about the data	
Timespan	2013:2022
Sources (Journals, Books, etc.)	170
Documents	675
Annual Growth Rate (%)	4.71
Document Average Age	4.03
Average Citations per Document	12.69
References	15,250
Document contents	
Keywords Plus (ID)	1270
Author’s Keywords (DE)	1213
Authors	
Authors	4658
Authors of Single-Authored Documents	3
Authors collaboration	
Single-Authored Documents	3
Co-Authors per Document	9.99
International Co-Authorships (%)	77.04
Document types	
Article	672
Article: Data Paper	1
Article: Early Access	2

**Table 2:** Top 10 major sources of research publications on VPDs in sub-Saharan Africa (2013–2022).

	Ranked by the Number of Publications		Source Local Impact				Ranked by the Number of Local Citations		
Rank	Source	No of Publications	H-Index	G-Index	M-Index	Total Citation	Rank	Source	No Local Citations
1	*Vaccine*	67	14	21	1.4	649	1	*Vaccine*	1443
2	*Clinical Infectious Diseases*	64	22	34	NA	1344	2	*Journal of Infectious Diseases*	1209
3	*PLos One*	55	15	22	1.5	640	3	*Clinical Infectious Diseases*	1036
4	*Pediatric Infectious Disease Journal*	42	13	20	1.3	509	4	*Lancet*	910
5	*Journal of Infectious Diseases*	27	13	25	1.3	635	5	*PLOS One*	890
6	*BMC Infectious Diseases*	26	10	18	1	346	6	*Pediatric Infectious Disease Journal*	877
7	*BMC Public Health*	16	7	12	0.7	168	7	*Journal of Clinical Microbiology*	518
8	*American Journal of Tropical Medicine and Hygiene*	13	6	8	0.75	72	8	*Lancet Infectious Diseases*	499
9	*Pan African Medical Journal*	12	4	5	0.67	33	9	*The New England Journal of Medicine*	471
10	*Human Vaccines & Immunotherapeutics*	10	5	9	0.5	96	10	*BMC Infectious Diseases*	351

## Data Availability

Data used in this review are publicly available in the literature.
